# Combined Anti-Bacterial Actions of Lincomycin and Freshly Prepared Silver Nanoparticles: Overcoming the Resistance to Antibiotics and Enhancement of the Bioactivity

**DOI:** 10.3390/antibiotics11121791

**Published:** 2022-12-10

**Authors:** Amna M. Abdul-Jabbar, Nehia N. Hussian, Hamdoon A. Mohammed, Ahmed Aljarbou, Naseem Akhtar, Riaz A. Khan

**Affiliations:** 1 Division of Biotechnology, Department of Applied Sciences, University of Technology, Baghdad 10066, Iraq; 2 Department of Medicinal Chemistry and Pharmacognosy, College of Pharmacy, Qassim University, Buraydah 51452, Saudi Arabia; 3 Department of Pharmacognosy and Medicinal Plants, Faculty of Pharmacy, Al-Azhar University, Cairo 11884, Egypt; 4 Department of Pharmaceutics, College of Pharmacy, Qassim University, Buraydah 51452, Saudi Arabia; 5 Department of Pharmaceutics, College of Dentistry and Pharmacy, Buraydah Private Colleges, P.O. Box 31717, Buraydah 51418, Saudi Arabia

**Keywords:** silver nanoparticles, lincomycin, anti-bacterial, anti-biofilm, synergistic effects, co-delivery, combined effect, anti-bacterial additive effect, gene expressions, *Bla* gene, *Bla_CTX-M-15_* gene

## Abstract

Bacterial drug resistance to antibiotics is growing globally at unprecedented levels, and strategies to overcome treatment deficiencies are continuously developing. In our approach, we utilized metal nanoparticles, silver nanoparticles (AgNPs), known for their wide spread and significant anti-bacterial actions, and the high-dose regimen of lincosamide antibiotic, lincomycin, to demonstrate the efficacy of the combined delivery concept in combating the bacterial resistance. The anti-bacterial actions of the AgNPs and the lincomycin as single entities and as part of the combined mixture of the AgNPs–lincomycin showed improved anti-bacterial biological activity in the *Bacillus cereus* and *Proteus mirabilis* microorganisms in comparison to the AgNPs and lincomycin alone. The comparison of the anti-biofilm formation tendency, minimum bactericidal concentration (MBC), and minimum inhibitory concentration (MIC) suggested additive effects of the AgNPs and lincomycin combination co-delivery. The AgNPs’ MIC at 100 μg/mL and MBC at 100 μg/mL for both *Bacillus cereus* and *Proteus mirabilis*, respectively, together with the AgNPs–lincomycin mixture MIC at 100 + 12.5 μg/mL for *Bacillus cereus* and 50 + 12.5 μg/mL for *Proteus mirabilis*, confirmed the efficacy of the mixture. The growth curve test showed that the AgNPs required 90 min to kill both bacterial isolates. The freshly prepared and well-characterized AgNPs, important for the antioxidant activity levels of the AgNPs material, showed radical scavenging potential that increased with the increasing concentrations. The DPPH’s best activity concentration, 100 μg/mL, which is also the best concentration exhibiting the highest anti-bacterial zone inhibition, was chosen for evaluating the combined effects of the antibiotic, lincomycin, and the AgNPs. Plausible genotoxic effects and the roles of AgNPs were observed through decreased *Bla* gene expressions in the *Bacillus cereus* and *Bla_CTX-M-15_* gene expressions in the *Proteus mirabilis*.

## 1. Introduction

Pathogenic bacteria have of late shown high resistance to known antibiotics of mild and strong natures at alarming proportions, and consequently the situation with anti-bacterial therapy has assumed distressing propositions. This has been further confounded by continuous discoveries of newer incidences of drug resistance by the microorganisms, which seems to be arising out of mutation and adaptability of the microorganisms to counteract the anti-bacterial agents’ actions for their survival. The unsuspected anti-bacterial agents have also started showing resistance in treatment to infections caused by certain specific and/or multiple kinds of microbial organisms. This has made the treatments of tuberculosis, gonorrhea, pneumonia, salmonellosis, and other food-borne diseases tough. The prevalence of treatment resistance to anti-bacterial agents has caused severe negative effects on animal and human health, their health costs, and the health systems. The multi-drug resistance (MDR) phenomenon is verifiable, and an existent global issue now. The antibiotics failures also become fatal, with bacterial anti-microbial resistance (AMR) being responsible for ~4.95 million mortalities every year, which is projected to rise to 10 million by 2050. The acronymic ESKAPE microorganisms, dealing with *Enterococcus faecium*, *Staphylococcus aureus*, *Klebsiella pneumoniae*, *Acinetobacter baumannii*, *Pseudomonas aeruginosa*, and *Enterobacter* spp. form the top priority status, which is currently posing the highest threat to humans [1].

There are several alternative methods available to control and circumvent bacterial AMR. The involvement of phage therapy and the use of bacteriocins, antibodies, and substitute antibiotics, as well as multiple antibiotics in treatments, are important. The development of newer antibiotic agents, the use of essential oils and other naturally sourced materials as single and combined multi-therapy regimen(s), and several molecules as part of bioactivity synergism have been proposed. The use of certain nanomaterials, especially metal and metal oxides as treatment additives, is also worth mentioning. These approaches are some of the constituent strategies forming part of the plan to combat AMR. These materials have exhibited explicit anti-bacterial activities by virtue of their chemo-pharmacological properties and have shown synergy as an additive material together with the antibiotic(s), thus playing major roles in inhibiting the occurrence/development of drug resistance by microorganisms. Towards an added edge, the synergistic action was observed to eliminate the need for high doses of antibiotics and to help minimize the unwanted side effects of the anti-bacterial agents [2]. However, additional factors to consider for developing effective strategies against MDR also include the cost, as well as capacity, and the approach’s effectiveness to manipulate the drug-resistant microbial species from leveraging towards the resistance.

Antibiotic resistance evolves through three general mechanisms: firstly, avoidance of the drug interactions with the target microbe; second, the antibiotic’s efflux from the interaction site cells; and, lastly, the modification(s) of the antibiotic’s chemical structure to make it ineffective. In this context, the role of certain enzymes, especially the β-lactamases, has assumed critical importance. Many previous reports have confirmed that certain microbial species produced extended-spectrum β-lactamases (ESBLs), the hydrolytic enzymes, that confer the microorganisms’ resistance towards the lactam-based antibiotics by their degrading act on the β-lactam ring of the antibiotics class, i.e., penicillins, cephalosporins, cephamycins, monobactams, carbapenems, and carbacephems. Some of the other approaches in structural modifications by the microorganisms also include acyl-transfer, phosphorylation, glycosylation, nucleotidylation, ribosylation, as well as thiol transfers [3,4,5].

*Bacillus* and *Proteus* spp. anti-bacterial resistance were attributed to β-lactamase actions, wherein the *Proteus* sp. acquired the resistance to ampicillin, a β-lactam antibiotic, through plasmid-mediated β-lactamase participation. The participation of the β-lactamase has been confirmed by the chromosomal β-lactamase expression. Notwithstanding the administration of clavulanic acid, a β-lactamase inhibitor, together with the β-lactam antibiotic to overcome bacterial resistance, trail has also failed. The resistance of *Bacillus cereus* to tetracycline antibiotics owing to the efflux action to eject the antibiotic from the cell, which prevents attaining the effective concentration of the drug in the cell to exhibit its anti-bacterial action, is accepted. Thus, the *B. cereus* is resistant to β-lactam antibiotics that included penicillin-G, amoxicillin/clavulanic acid, as well as tetracycline. The *B. cereus* is also highly sensitive to lincomycin, followed by erythromycin. The *P. mirabilis* showed high sensitivity against trimethoprim-sulfamethoxazole, and cefotaxime, as well as was resistant to lincomycin, amikacin, and amoxicillin/clavulanic acid. Genetically, on the other hand, previous reports have also shown that the presence of *tet* genes in *B. cereus* contributes to their resistance to anti-bacterial agents [5]. In this scenario, the role of other molecular templates, metal and metal oxide compounds at nano and micro-levels, as well as other biochemical and chemical entities, as additive and synergic partners, assumes utmost importance.

Silver nanoparticles (AgNPs) have confirmed anti-bacterial efficacy against both the Gram-positive and Gram-negative bacterial strains [6,7]. The use of AgNPs as an anti-bacterial agent has provided significant toxicity against microorganisms at low doses. The nanoscale size, shape, and charge of the AgNPs have facilitated their high permeability across the cellular membranes of the microorganisms [8] and exhibited anti-bacterial actions through multiple mechanisms [9]. There are also reports to show the nanoparticle’s genotoxic features, causing the DNA and RNA damage, their interference with the genetic materials’ replication processes, as well as hindering the expressions of the genetic information in cells, together with their mutagenic effects. These modes of action can be related to the direct influence of NPs, and metal ions, as well as the indirect effects of the Reactive Oxygen Species (ROS)-mediated activities that seemed to prompt the Save Our Soul (SOS) reactions in the bacterial cells [9].

Lincomycin, a naturally sourced lincosamide antibiotic obtained from the actinomycete species, *Streptomyces lincolnensis*, is used to treat penicillin-allergic patients and drug-resistant bacterial infections of multiple types. It has been rendered less bioactive as an anti-bacterial agent. A 600 mg dose of lincomycin injected intravenously over two hours resulted in an average C_max_ of 15.9 g/mL, but the same dose when injected intramuscularly resulted in an average C_max_ value of 11.6 g/mL after 1 h (hour) of drug administration. The biological half-life of 5.4 ± 1.0 h of the drug for the susceptible Gram-positive microorganisms has complicated the dose demand, which is very high on a 600 mg scale for the single infusion [10]. This has prompted us to look into the lincomycin bioactivity profile and ascertain ways to overcome the activity fall. However, the restricted Gram-positive anti-bacterial activity and continuously developing resistance to the drug have also lowered the therapeutic efficacy of the drug. The situation has been further complicated by the high hepatic and renal toxicities of the drug. Moreover, dialysis has been rendered ineffective in removing the excessive drug contents from systemic circulation. Nonetheless, the blood–brain barrier (BBB) permeability of the drug has been found inadequate to treat meningitis [11,12,13]. In this context, the use of AgNPs, known for their facilitated anti-microbial activity, against both the Gram-positive and Gram-negative bacterial strains, assumes significance. The AgNPs’ physico-chemical properties, i.e., small particle size with large surface area, which enabled them to interact and/or penetrate the cell walls and membrane, have affected the intracellular components, leading to bacterial cell death. This has been achieved through different mechanisms, which include inhibition of the metabolic pathway, protein synthesis, interference with nucleic acid (DNA) synthesis, and cell wall disruptions [14]. The surface-area-to-volume ratio becomes larger when the particle size is small, and this was translated into the drug being closer to the surface of the nanoparticles, as compared with the larger-sized particles. The faster reach of the drug and the nanoparticles within or in the proximity of the cell catapulted to better bioactivity [15].

The current study aimed to improve the anti-bacterial efficacy of the lincomycin antibiotic in the presence of AgNPs and observe the roles of the AgNPs’ anti-oxidant potential, as well as the effects of the AgNPs delivery on the genes’ expressions of the bacterial isolates, *Bacillus cereus*, and *Proteus mirabilis* at the genetic involvement levels.

## 2. Materials and Methods

### 2.1. Materials Procurement

Chemicals and reagents utilized for the preparations of AgNPs, including silver nitrate (AgNO_3_, MW: 169.87 amu), tri-sodium citrate di-hydrate (Na_3_C_6_H_5_O_7_, MW 294.10 amu), and sodium dodecyl sulfate (SDS, C_12_H_25_NaSO_4_, MW 288.38 amu), were purchased from BDH, West Yorkshire, England. Deionized water was obtained from Baghdad Company, Baghdad, Iraq. Lincomycin (C_18_H_34_N_2_O_6_S, MW 406.538 amu) was purchased from Livealth Biopharma, MS, India. The culture media MacConkey agar, agar base media (MYP), Muller–Hinton agar, tryptic soy broth (TSB), and brain–heart infusion broth were obtained from Mast, Liverpool, England. Phosphate buffer solution (PBS), crystal violet, and 1,1-diphenyl-2-picryl-hydrazyl (DPPH, MW 394.32 amu) were purchased from Sigma-Aldrich, Darmstadt, Germany. Antibiotic disks were obtained from Liofilchem, Roseto degli Abruzzi, Italy. The kits were purchased from TransGen Biotech, Beijing, China, and were used according to the manufacturer’s specifications and instructions.

### 2.2. Isolation and Identification of Bacterial Isolates

Bacterial isolates, *B. cereus*, and *P. mirabilis* were locally obtained and identified by morphological characterizations on blood agar. For *P. mirabilis* MacConkey agar was used, and for *B. cereus*, the selective agar base media (MYP) was utilized, followed by microscopic characterizations, biochemical tests, and the use of the Vitek-2 system.

### 2.3. Antibiotics Susceptibility Test

Bacterial isolates’ sensitivity to antibiotics was determined by the Kirby–Bauer disk diffusion method. The tested antibiotics included Amikacin (AK), Amoxicillin/clavulanic acid (AMC), Cefotaxime (CTX), Erythromycin (E), Lincomycin (MY), Penicillin (P), Tetracycline (TE), and Trimethoprim-sulfamethoxazole (STX) (CLSI 2021). The antibiotics were combined with AgNPs and tested against the bacterial isolates. The FI percent was calculated as Fold Increase (%) = (b − a)/a × 100, where a and b denoted the zones of inhibitions for the antibiotic alone and antibiotic with AgNPs, respectively [16,17].

### 2.4. Synthesis of AgNPs

The chemical reduction method was used to prepare AgNPs. Silver nitrate (0.0849 g) was dissolved in 100 mL of deionized water. At 80 °C, trisodium citrate dihydrate (0.0103 g), and sodium dodecyl sulfate (0.0144 g) were mixed in 100 mL of deionized water. The two mixtures were added dropwise over 30 min under continuous stirring, and the reaction was continued at 80 ° C for 4 h to ensure the completion, which showed yellow color. The produced AgNPs were cooled, collected in an amber bottle, and refrigerated [18].

### 2.5. Preparation of Lincomycin Antibiotic Stock Solution

A stock solution of lincomycin was prepared with a final concentration of 15 µg/mL by mixing 0.2 mL from the lincomycin vial, and the volume was raised to 1 mL by the addition of distilled water.

### 2.6. Characterization of Prepared AgNPs

UV-Vis spectroscopy (PerkinElmer, Waltham, MA, USA) was used to detect the absorption maxima λ_max_ of the synthesized AgNPs, Fourier Transform-infrared spectroscopy (FT-IR, PerkinElmer, Waltham, MA, USA) was used to record the IR spectrum, and X-ray diffraction (XRD) (Haoyuan, Zhejiang, China) instrument was used to detect the crystallinity of AgNPs. The dynamic light scattering (DLS, Brookhaven, NY, USA) was used to determine the NP size distributions, energy-dispersive X-ray (EDX) and microscopic analyses were performed using field-emission scanning electron microscopy (FE-SEM, Tescan Orsay Holding, Brno, Czech Republic), and transmission electron microscopy (TEM, Zeiss, Jena, Germany) was performed to detect the morphological features.

### 2.7. Combined Effects of the Co-Delivered AgNPs–Lincomycin Mixture

AgNPs (100 µg/mL) was mixed with lincomycin to a final concentration of 15 µg/mL. A total of 0.2 mL of lincomycin in 0.8 mL of AgNPs was mixed and well-homogenized on a stirrer, and the resultant solution was kept in dark at room temperature [19]. The well-diffusion method was used to test the anti-bacterial activity of the lincomycin, AgNPs (100, 50, 25, and 12.5 μg/mL), and the mixture of AgNPs–lincomycin.

### 2.8. Biofilm Formation Test

The biofilm formation was detected before and after the treatments with AgNPs (100, 50, 25, and 12.5 μg/mL), lincomycin alone (15 µg/mL), and an AgNPs–lincomycin mixture [20]. In test tubes, a loop-full of test isolates were inoculated in 5 mL of tryptone soya broth, and for 24 h the tubes were incubated at 37 °C, later decanted, washed with phosphate buffer saline (pH 7.4) and left to dry. The tubes were stained with crystal violet, excess stains were washed with deionized water, and the tubes were dried upside down.

### 2.9. Evaluation of the MIC, and MBC

The minimum inhibitory concentration (MIC) and minimum bactericidal concentration (MBC) were determined for the AgNPs, and AgNPs–lincomycin mixture against *B. cereus* and *P. mirabilis* strains [21,22]. In brief, 0.8 mL of the brain–heart infusion broth medium was added to test tubes, then 0.1 mL of (12.5, 25, 50, 100 µg/mL) of each solution was added. Then, 0.1 mL of the suspension for each tested bacteria, *B. cereus*, and *P. mirabilis* were added after being compared with a standard McFarland tube, The tubes were well shaken and incubated for 24 h at 37 °C, and then, based on turbidity formations, the results were recorded. Mueller–Hinton agar medium was inoculum with 100 µL of the mixture and incubated at 37 °C for 24 h, depending on whether there was growth (+) or no growth (−), the result is reported.

### 2.10. DPPH (1, 1-Diphenyl-2-picryl-hydrazyl) Assays

The antioxidant activity of AgNPs and AgNPs–lincomycin mixture was evaluated with stable DPPH radicals. The scavenging activities of AgNPs and AgNPs–lincomycin were evaluated at concentrations 12.5, 25, 50, and 100 μg/mL. The concentration of ascorbic acid (+ ve control) was 10 µg/mL. Further, 750 μL of each sample was mixed with 750 μL of DPPH solution prepared by dissolving 0.02 g DPPH in 50 mL methanol. Then, each sample was stored for a half-hour at 37 °C away from light. Optical density (OD) was measured at 517 nm wavelength [7], and the activity was calculated according to the following equation:

(1) $$\mathrm{Antioxidant}\;\mathrm{activity}\;=\frac{\mathrm{OD}\;\mathrm{control}\;-\;\mathrm{OD}\;\mathrm{sample}\;}{\;\mathrm{OD}\;\mathrm{control}\;}\times \%\;100$$

### 2.11. Growth Curve Tests

AgNPs’ (100 µg/mL) activity against growths of *B. cereus* and *P. mirabilis* was determined [23]. The 0.1 mL of AgNPs and 0.1 mL of bacterial suspensions were added to 10 mL of nutrient broth, and 0.1 mL was spread on the Mueller–Hinton agar plate, incubated at 37 °C for 24 h, and the viable bacterial cells were observed at 0, 30, 60, and 90 min intervals.

### 2.12. Analyses of Genes Expression Levels

Genic expression levels of the two antibiotic resistance genes, β-lactamase producing (*Bla*) in *B. cereus* [24], and (*Bla_CTX-M-15_*) in the *P. mirabilis* were examined [25,26]. First, the cells’ total RNA (treated and untreated with AgNPs) was extracted using an RNA extraction kit (TransGen Biotech, Zol UpPlus RNA Kit) according to the manufacturer’s instructions. Subsequently, cDNA synthesis was performed (EasyScript^®^ One-Step gDNA Removal and cDNA Synthesis SuperMix kit), also according to the manufacturer’s instructions. The cDNA was amplified using TransStart^®^ Top Green qPCR Super Mix (TransGen, Biotech. AQ131-01) kit. For the gene expression levels of *Bla* for *B. cereus*, the primers, Forward 5′-CATTGCAAGTTGAAGCGAAA-3′, and Reverse 5′-TGTCCCGTAACTTCCAGCTC-3′ were used. For the *Bla_CTX-M-15_* gene for the *P. mirabilis*, the primers used were Forward 5′-ACGCTGTTGTTAGGAAGTG-3′ and Reverse 5′-TTGAGGCTGGGTGAAGT-3′ with Real-Time PCR program as 94 °C (the 30 s), 94 °C (5 s), 52 °C (15 s), and 72 °C (20 s) for 35 cycles. The primer sequences of *Bla* and *Bla_CTX-M-15_* genes were designed and synthesized by (Alpha DNA, Montreal, QB, Canada). Finally, “2^−ΔCt^” was assigned to the expression, which determined the changes in the relative folds. The results were expressed as a fold change in the levels of expression of the study gene relative to the study gene in control, and a housekeeping gene [27].

#### 2.12.1. Extraction of Total RNA

Total RNA was extracted from bacterial strains treated with AgNPs, and the untreated bacteria (used as controls) by using an RNA extraction kit (TransZol UpPlus RNA Kit), according to the manufacturer’s instructions, and then the concentration and purity of the extracted RNA were measured using 2000c Nano-drop spectrophotometer (Thermo Fisher Scientific, Carlsbad, CA, USA).

#### 2.12.2. Reverse Transcription (RT)-PCR

The EasyScript^®^ One-Step gDNA Removal and cDNA Synthesis SuperMix kits were used to reverse transcribe the total RNA to cDNA. The process was carried out with a volume reaction of 20 µL following the manufacturer’s protocol, and 4 µL of total RNA needed to be reverse transcribed. The cDNA was obtained, then it was amplified by PCR with thermal cycle steps purposed for the primer annealing, DNA polymerization, and enzyme deactivation, respectively, as exhibited in Table 1. This yielded multiple copies of cDNA and was then used as the template for quantitative real-time PCR using the Power SYBR Green Master Mix.

#### 2.12.3. Quantitative Real-Time PCR

The QIAGEN Rotor gene Q Real-time PCR System (Germany) was used to perform the qRT-PCR. To evaluate the levels of expressions, and fold changes of the *Bla* and *Bla_CTX-M-15_* genes, the TransStart^®^ Top Green qPCR Super-Mix and threshold cycle (Ct) measurements were used. The thermal profiles and following optimal cycles were applied to program the cycling protocol (Table 2).

### 2.13. Statistical Analyses

GraphPad Prism 7.0^®^ software (GraphPad Software, San Diego, CA, USA) was used. The mean ± SD (standard deviations) values were compared, using the least significant difference (LSD).

## 3. Results and Discussion

### 3.1. Preparation and Characterization of AgNPs

**Figure 1:**
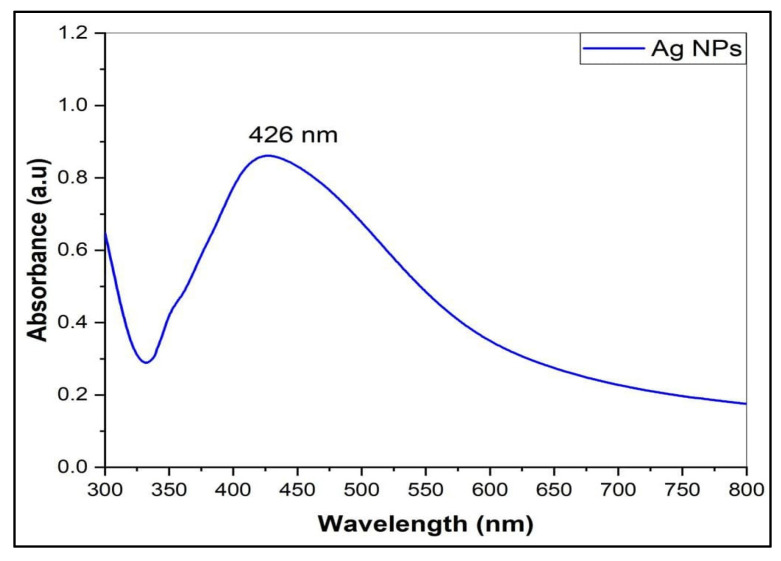
The UV-Visible spectrum of AgNPs.

Silver nanoparticles (AgNPs) were synthesized by the chemical reduction method. The AgNO_3_ was reacted with trisodium citrate dihydrate (TSC) and sodium dodecyl sulfate (SDS) as the emulsifier to control the size and provide stability to the prepared silver nanoparticles colloid. The prepared AgNPs were characterized by their UV-Visible spectrometric absorbance in the UV-Vis (Ultra Violet-Visible) range of 200–800 nm. Figure 1 shows the absorption maxima, λ_max_ at 426 nm, and the free electrons in the metal nanoparticles yielded the Surface Plasmon Resonance (SPR) absorption band; the absorption peak pattern and the color of the prepared AgNPs (yellow) indicated the size and shape of the NPs [28].

**Figure 2:**
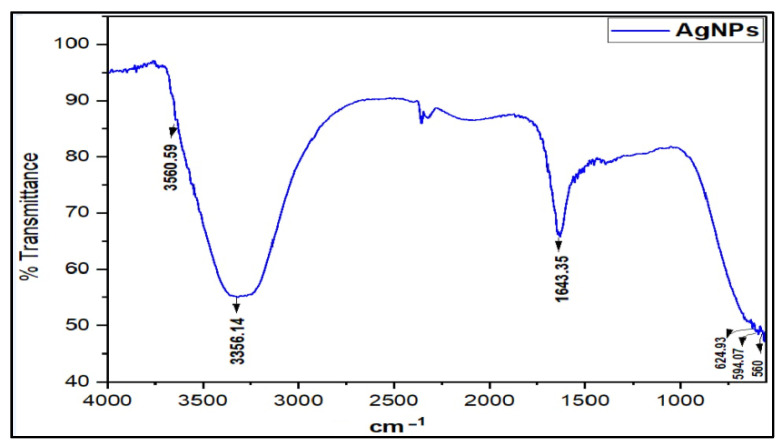
FT-IR absorption spectrum of AgNPs.

The Fourier Transform Infra-Red (FT-IR) spectrum of the prepared AgNPs was recorded to determine the presence of functional groups. The characteristic IR absorption major broad peak at ν_max_ 3356.14 cm^−1^ accounted for the presence of OH, and the sharp peak at 1643.35 cm^−1^ attributed to the carbonyl (-C=O) stretching, together with minor peaks at 2350.06, 624.93, and 594.07 cm^−1^, which agreed with the absorption values of the previous studies on the citrate-capped AgNPs characterization [7,29] (Figure 2). Moreover, the AgNPs IR spectrum differed from the starting material, silver nitrate, which exhibits a characteristically strong metal nitrate peak and is devoid of the hydroxyl function peaks. The AgNPs spectrum also differed with the SDS and TSC, the emulsifier and capping agent used in the synthesis, respectively. The synthesis of the AgNPs was also confirmed by other analyses and morphological observations.

**Figure 3:**
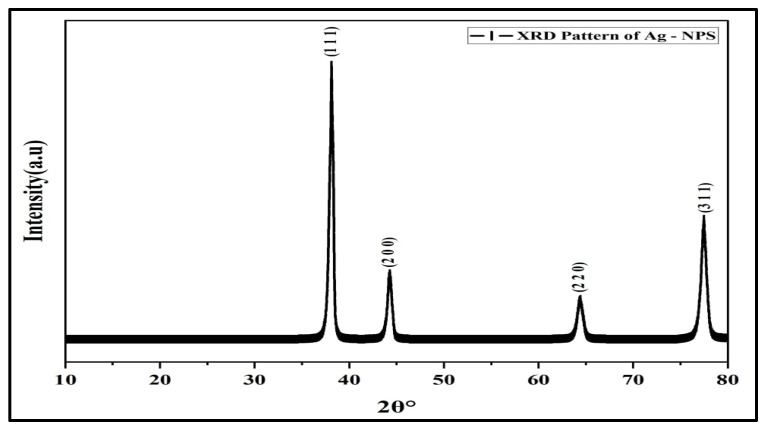
XRD pattern of AgNPs.

The X-Ray Diffraction (XRD) patterns of characteristic sharp peaks for AgNPs were located at 2θ at 38.117°, 44.279°, 64.428°, and 77.475°, and indexed to (111), (200), (220), and (311) planes, respectively, belonging to the face-centered cubic (FCC) crystalline structure of the silver (JCPDS, file No. 04-0783) (Figure 3). The finding agreed with a previous study [30]. The average crystalline size was ~34.28 nm, which was calculated from Scherrer’s Equation [31].

**Figure 4:**
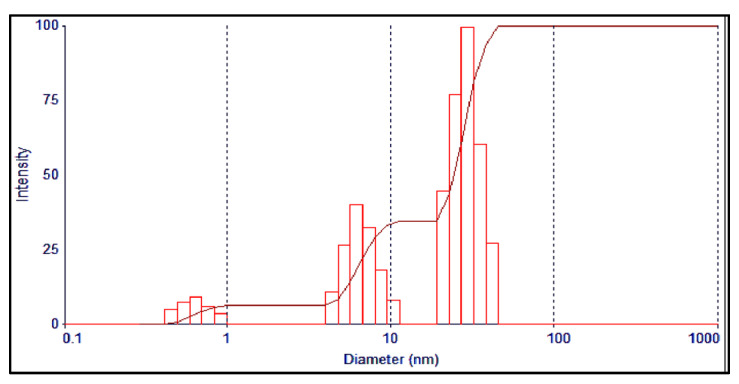
DLS analysis of the AgNPs.

**Figure 5:**
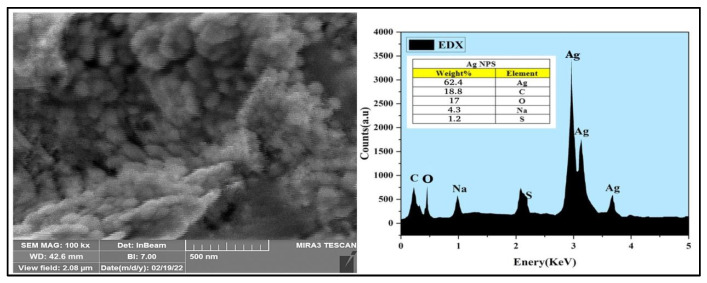
SEM and EDX analyses of the AgNPs.

The Dynamic Light Scattering (DLS) analysis of the AgNPs’ showed the average particle size as 53 nm, and the PDI (polydispersity index) at 0.320 (Figure 4). The SEM (Scanning Electron Microscopy) analysis of the AgNPs showed the morphological features and structural details, which have a smooth surface and spherical shapes, with some agglomeration and aggregation of the nanoparticles. The mean size ranged between 31.26 to 67.78 nm (Figure 5), which agreed with the size observed through TEM (Tunneling Electron Microscope) and the inputs from Scherer’s equation as predicted from the X-ray analysis observations [32].

The Energy-dispersive X-ray (EDX) analysis demonstrated the weight percentage of silver in the AgNPs and was at 62.4% of the overall components of the sample, with just trace amounts of the chemicals present, which were utilized to synthesize the AgNPs (carbon 18.8%, oxygen 17%, sulfur 1.2%, and sodium 4.3%) in the AgNPs.

**Figure 6:**
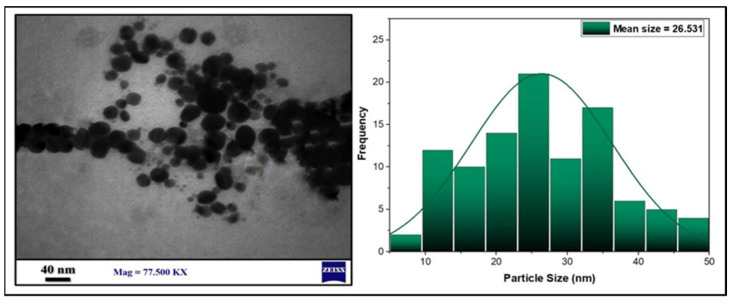
TEM images and histogram of the AgNPs particle size distributions.

To further analyze the size and its distributions, as well as the morphology of the synthesized AgNPs, a 77,500× magnification was used for TEM analysis. The TEM demonstrated that most of the prepared AgNPs have small spherical shapes with an average mean size of 26.531 nm, as also exhibited in the histogram. Then, ImageJ software (Java 1.8.0, Gaithersburg, MD, USA) was used to determine the particles’ diameters (Figure 6).

However, the storage conditions and storing period of the AgNPs alter their physicochemical properties, including their sizes, shapes, particles aggregations, formation of insoluble materials, and release of the silver ions, together with the formation of new silver compounds, e.g., Ag_2_O. Leaching of the AgNPs’ capping material in the colloidal solution also occurs. The changes in nanoscale properties also affect the potential biological actions of the AgNPs [33]. To keep the properties relevant to the use of AgNPs, fresh preparation of the AgNPs was used for all experimental purposes during the study.

### 3.2. Plausible Mechanism of AgNPs Conjugation with Lincomycin

**Figure 7:**
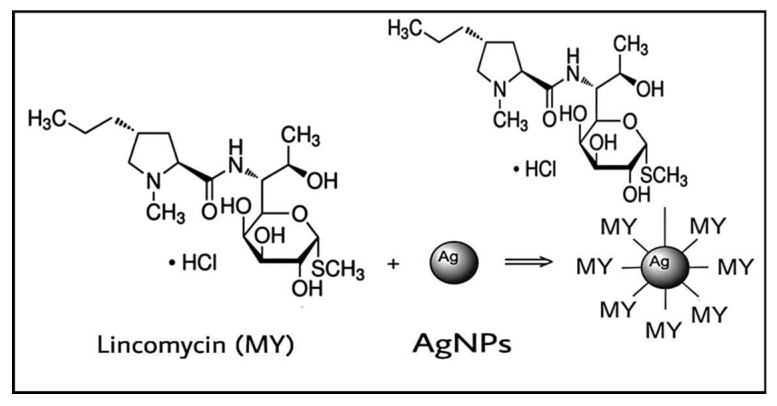
Chemical interaction of lincomycin with AgNPs.

Lincomycin–AgNPs were mixed (Figure 7), and their effects as combined anti-microbial agents were studied. The citrate (TSC)-capped, negativity-charged AgNPs were mixed with the lincomycin, which contained the thio-methyl (-SCH_3_) group. It is suggested that the lincomycin adsorption on the AgNP surface through electrostatic interaction may have provided a certain degree of protonations of the tertiary amine as (-N^+^<), secondary amine as (-NH^+^-), and the ether group as (-C-O^+^-C-), which seemed to stabilize the mixture with the electrostatically negatively charged AgNPs [34].

### 3.3. Culture Identifications

**Figure 8:**
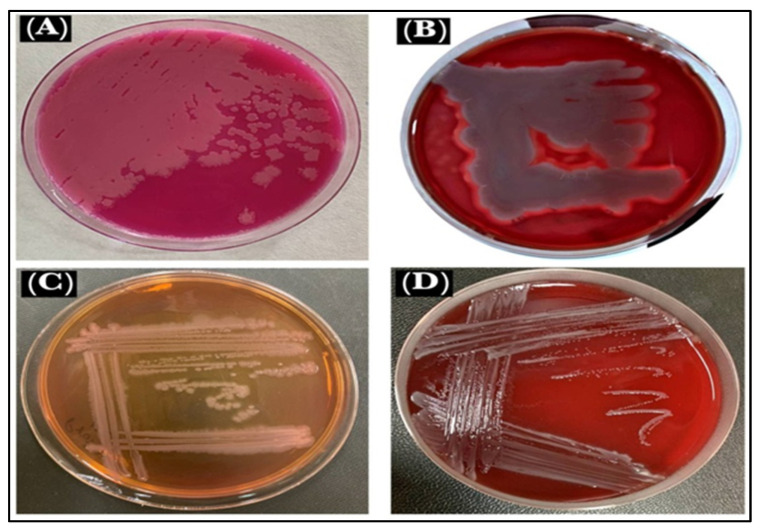
Microbe views: (**A**). *B. cereus* on MYP, (**B**). *B. cereus* on blood agar, (**C**). *P. mirabilis* on MacConkey agar, and (**D**). *P. mirabilis* on blood agar.

The *B. cereus* showed pink identical colonies surrounded by a white zone on *B. cereus* selective agar base media (MYP), the medium that differentiated the *B. cereus* from other bacteria. This is based on mannitol fermentation, lecithinase activity, and resistance to polymyxins molecules [35]. On blood agar under aerobic conditions at 37 °C, the *B. cereus* colonies morphology appeared as large, gray, and opaque with a rough-matted surface surrounded by a wide zone of β-hemolysis [36]. The *P. mirabilis* culture on MacConkey agar showed pale and colorless, with smooth colonies, which confirmed that it was a non-lactose fermenter, and when cultured on Blood agar appeared in grey, as spreader colonies thereby confirming the swarming motility and β-hemolysis of the *P. mirabilis* (Figure 8).

### 3.4. Microscopic Characterizations

**Figure 9:**
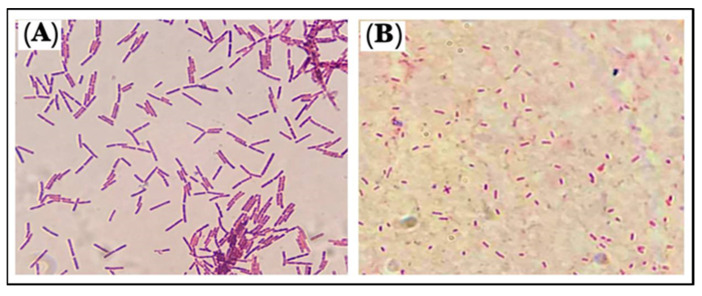
Morphology of the microbes: (**A**). *B. cereus* and (**B**). *P. mirabilis*.

The used Gram stains were examined under a light-based microscope. *B. cereus*, a Gram-positive strain, were rod-shaped bacilli with rounded ends, non-capsulated in structure, and were either single-rod or short-chain structures with a purple color. The *P. mirabilis*, Gram-negative strain, were rod-shaped, pink-colored, and motile by flagella (Figure 9). The Gram-positive bacteria appeared purple because the stain, crystal violet, was used, whereas the Gram-negative bacteria appeared pink because the cells had a thin layer of peptidoglycan, and the crystal violet was washed off when ethanol was added to them [37].

### 3.5. Antibiotic Susceptibility Tests

Antibiotic susceptibility tests were run for several antibiotics against both microorganisms. The susceptibility test was also run for the combined delivery of AgNPs and lincomycin. The results are summarized in Table 3.

The *B. cereus* is generally resistant to β-lactam antibiotics that included penicillin-G (P), amoxicillin/clavulanic acid (AMC), as well as tetracycline (TE). The *B. cereus* is also highly sensitive to lincomycin (MY), followed by erythromycin (E). The *P. mirabilis* showed high sensitivity against trimethoprim-sulfamethoxazole (STX), and cefotaxime (CTX), as well as also showed resistance to lincomycin (MY), amikacin (AK), and amoxicillin/clavulanic acid (AMC). The same bacteria were also sensitive to the AgNPs–antibiotic mixture. AgNPs were tested against *B. cereus*, and *P. mirabilis* at different concentrations (12.5, 25, 50, and 100 µg/mL) to determine their anti-bacterial activities and showed varying results. The exhibited zone of inhibition confirmed that the highest anti-bacterial effects of the AgNPs were at 100 μg/mL concentration, and this effect gradually decreased with the decrease in the AgNPs concentrations, thereby exhibiting a dose-dependent relationship. The interactions between the sulfur and phosphorus-containing proteins of the bacterial cell wall and the AgNPs may have led to the disruption of the cell wall. In addition, the anti-bacterial activity exhibited by the AgNPs’ may be due to the AgNPs’ presence at the bacterial cell walls’ surfaces, primarily owing to the electrostatic interactions between the microorganisms and the AgNPs, which may have played a part [38]. Moreover, the AgNPs’ anti-bacterial potential is also said to be influenced by the nanoparticles’ size (as well as charge) and the bacterial cell wall’s structure. The AgNPs have shown higher anti-microbial activity against the Gram-negative strain, as compared to the Gram-positive strains of the bacteria, which are unrelated and independent of the bacterial resistance levels. This can be explained by variations in the two strains’ cell wall structures. For Gram-positive bacteria, the cell wall is composed of a thick layer of peptidoglycan, while in Gram-negative bacteria, it mainly consisted of lipopolysaccharide, followed by a thin layer of peptidoglycan. The metallic nanoparticles provide a larger surface area in contact with the bacterial cell wall at the surface due to their size and the surface ratio on the nanoscale [39].

**Figure 10:**
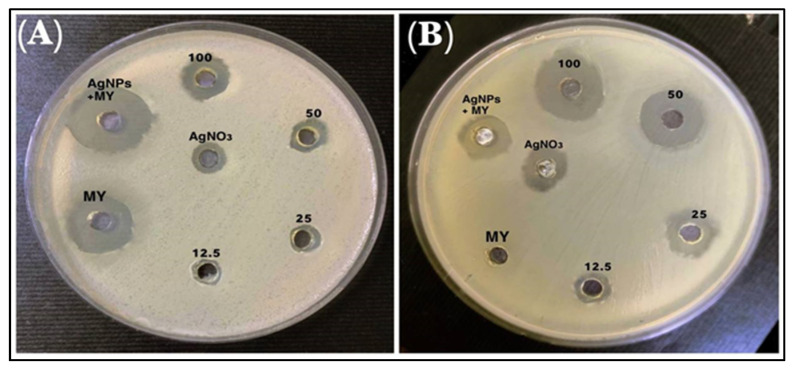
The inhibitory effects of different concentrations of AgNO_3_, AgNPs, lincomycin (MY), and AgNPs–lincomycin on (**A**) *B. cereus* and (**B**) *P. mirabilis*.

The inhibition zone diameter for lincomycin alone and the mixture of AgNPs and lincomycin showed an increase in the inhibition zone; therefore, the anti-bacterial efficacy of the lincomycin increased synergistically with AgNPs against *P. mirabilis*. As for the *B. cereus*, the diameter of the inhibition zone increased significantly (Figure 10, Table 4). The lincomycin mechanism of action on bacteria is understood to have interfered with the metabolic pathways in the bacteria, which leads to inhibiting the synthesis of microbial protein. The encoding of the 23S rRNA of the 50S subunit and imitation of the intermediate formed in the initial phases of the elongation cycle also contribute to this [40]. On the other hand, microbial resistance to lincosamide antibiotics is pursued by different methods, which include (i) by modifying the target region to prevent the antibiotic from binding to the ribosomal target through methylation, or mutation, (ii) by drug inactivation, and (iii) through efflux of the antibiotic. These mechanisms are also found in macrolide antibiotics and some of the lincosamide class antibiotics, as also discussed earlier.

At the clinical level, the antibiotic’s mechanism of action in pathogenic microorganisms varies in proportion to the medical complications, and the disease incidence. Alteration of the ribosomal target addresses broad-spectrum resistance to lincosamides and macrolides, while inactivation and efflux only affect some of these antibiotic molecules [41]. This study shows that the action of AgNPs–lincomycin enhanced the anti-bacterial activity of the antibiotic against the microorganisms. It offered the potential to reduce the adverse effects of the excessive antibiotic dose, and end the development of bacterial resistance [42,43]. The results also show that the lincomycin inhibited the biofilm formation in *B. cereus*, and as for the *P. mirabilis*, the strength of biofilm formation was also slightly reduced, but to a lesser extent as compared with the negative control. These results also indicate that the biofilm formation was dependent on biofilm-related genes, and their expressed proteins that underwent bacterial control and its eventual availability and use in biofilm formation with sub-MIC of the antibiotic were noted [44]. The contribution of the neutralization of the bonding agent materials involved in biofilm development is also known [43]. The effectiveness of the AgNPs–lincomycin mixture in inhibiting the formation of biofilm by both the microorganisms used in this study also suggested the combined action of the lincomycin antibiotic and the AgNPs. The physicochemical characteristics, including size, shape, and chemical composition, of the prepared AgNPs, have a significant impact on their anti-bacterial activity. According to a study by Mohsen and his coworkers, compared to spherical-shaped AgNPs, the prism-shaped AgNPs significantly inhibited the growth of both Gram-positive (*S. aureus*) and Gram-negative (*E. coli*) bacterial strains. Due to their effective anti-bacterial features, the nano-prisms’ multiple sharp edges and vertexes made it easier for the AgNPs nano-formulation to penetrate the bacterial cell walls and cause cell wall destruction [45]. Previous studies have also reported the potential impacts of AgNPs on human cells. For in vitro experiments in various cell lines, the AgNPs have shown cytotoxic effects depending upon their size, shape, coating, dosage, and cell types. Both the immediate and distant organs were affected by the routes of administration, particle size, coating material, dose, duration of exposure, and endpoint measurement times [46].

### 3.6. Biofilms Formations

**Figure 11:**
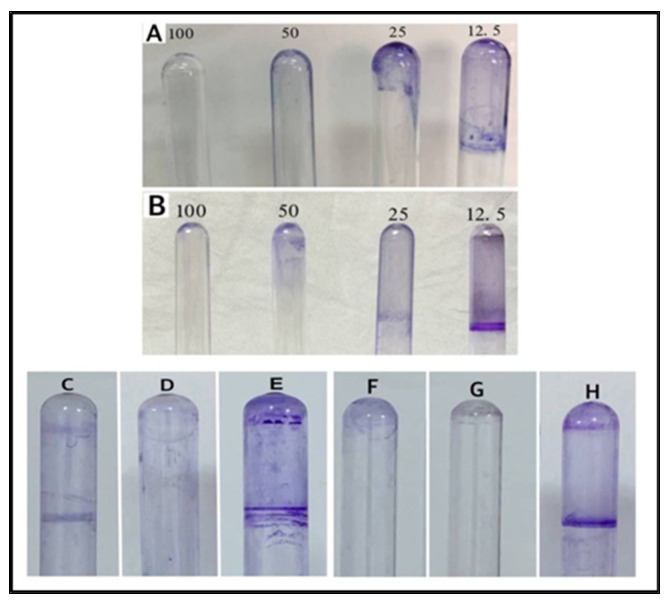
Biofilm formations: (**A**) AgNPs on *B. cereus*, (**B**) AgNPs on *P. mirabilis*, (**C**) lincomycin on *P. mirabilis*, (**D**) AgNPs–lincomycin on *P. mirabilis*, (**E**) positive control of *P. mirabilis*, (**F**) lincomycin on *B. cereus*, (**G**) AgNPs–lincomycin on *B. cereus*, and (**H**) positive control of *B. cereus*.

Biofilms are accumulations of bacteria that are adhered to the surfaces and embedded in a self-produced matrix. The matrix of biofilm contains substances, such as proteins, polysaccharides, and extra-cellular DNA, which protect bacteria from harsh environmental conditions and provides resistance to human immunity. The film can also withstand a variety of chemotherapeutic agents. Since infections produced by bacteria that form a biofilm are hard to treat, there is a need to search for new and novel biofilm inhibitors [42]. The effects of different concentrations of AgNPs (12.5, 25, 50, and 100 μg/mL) on bacterial isolates, used in this study, show that the AgNPs were able to inhibit the formation of biofilm by both the *B. cereus* and *P. mirabilis* at 100 μg/mL concentrations. The biofilm formations were uninhibited at low concentrations (12.5 μg/mL) of the AgNPs against both the bacterial isolates (Figure 11). Furthermore, the primary step for biofilm formation is the adhesion of bacteria to the host surface. The AgNPs’ adhesion on the bacterial cell wall surfaces and their disruption of the permeability, and metabolic pathway leading to cell death is well known [9]. Nonetheless, this inhibition of biofilm formation is of immense practical importance, specifically in the fight against drug-resistant pathogenic microbes, together with the use of a higher concentration of the AgNPs, which facilitated the anti-bacterial activity. The observation also provided a much-needed rationale for the efficacy of the co-delivery of antibiotics and the nanoparticles. To explain these results, two hypotheses have been suggested: (i) the Ag ions may inhibit the development of biofilms in bacteria by entering the bacterial cell and interfering with the proteins and enzymes necessary for microbial adhesion [47]; (ii) the AgNPs may weaken the development of biofilms by preventing the production of exogenous polysaccharides (exopolysaccharides). The NPs prevent the bacterial cell wall’s layers of polysaccharides from forming a biofilm by entering the water channels (aquaporin) that carry water and nutrients [48].

### 3.7. Measurements of the MIC and MBC of AgNPs and AgNPs–Lincomycin Mixture

**Figure 12:**
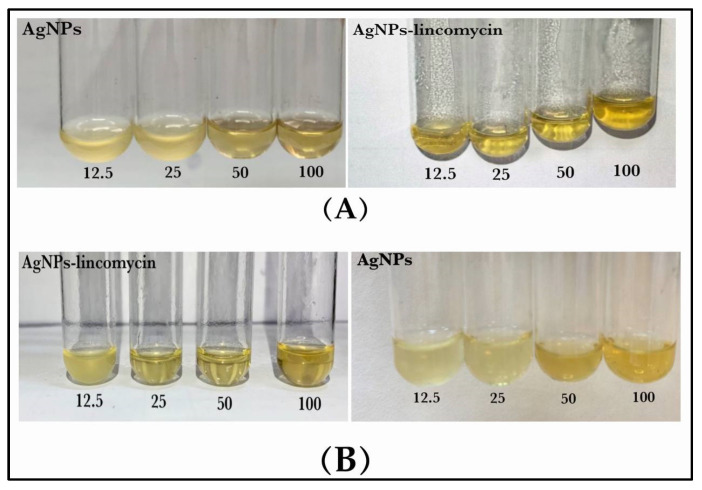
MIC and MBC of the AgNPs and AgNPs–lincomycin against bacterial isolate: (**A**) *B. cereus* and (**B**) *P. mirabilis*.

Turbidity assays were performed to determine the effects of AgNPs and AgNPs–lincomycin according to the bacterial growth of the isolates used in this study using the broth media. The results of the MIC and MBC are shown in Table 5 and Figure 12. These results exhibited that the MIC of the AgNPs–lincomycin for *B. cereus* at 12.5 μg/mL was lower than for *P. mirabilis* MIC of AgNPs–lincomycin, which was at 25 μg/mL. Through the tested bacteria, the lowest value of the MIC was obtained for the AgNPs–lincomycin that showed high anti-bacterial activity with a decrease in the MIC, as compared with AgNPs alone. To determine the MBC value, which is the concentration at which the tested bacteria were killed, solid culture media were used, which confirmed the absence of bacterial growth [7,49]. The MBC values were higher than the MIC values. Accordingly, the MBC of the AgNPs alone for the *B. cereus* and *P. mirabilis* were at 100 μg/mL. As for the AgNPs–lincomycin, the MBC of *B. cereus* was at 25 μg/mL and for *P. mirabilis* it was at 50 μg/mL. The reactive oxygen species (ROS), along with free radicals, seemed to be produced by the AgNPs, which damaged the bacterial cell wall and also inhibited the respiratory enzymes. In general, the main action of the AgNPs in an aqueous microenvironment seemed to be that the silver nanoparticles continuously released silver ions. The structure of the cell wall, the specific enzymes produced by the bacteria, and the concentrations of the bacterial suspension all had an impact on the MBC value [50].

### 3.8. Growth Curve Test

**Figure 13:**
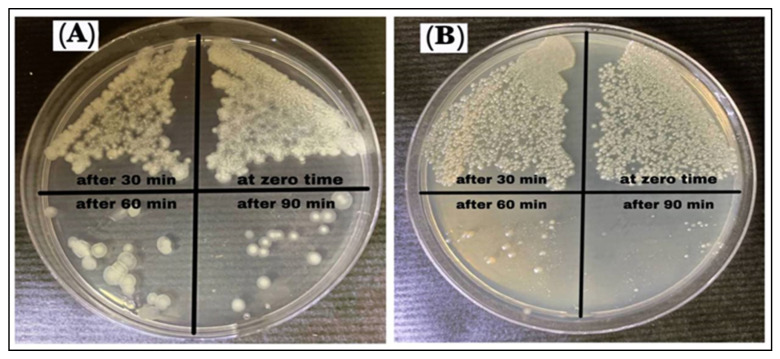
Growth curve assay of AgNPs against (**A**) *B. cereus* and (**B**) *P. mirabilis*.

The growth curve test was used to estimate the anti-bacterial activity of the AgNPs (100 µg/mL), which showed that the time required was 90 min to kill the *B. cereus*, and *P. mirabilis* bacterium, as shown in Table 6 and Figure 13. It was observed that at zero time, there was no effect on the bacterial growth, while after 30 min the growth of bacteria began to be inhibited. After 60 min the growth inhibition significantly increased. At 90 min, a little bacteria growth was observed in a few colonies, and this is related to the interactions of the AgNPs with the bacterial cell membrane. It is suggested that the sulfur and phosphorus-containing entities contained within the components of the cell walls are the favorite sites for the AgNPs, which leads to bacterial cell breakdown and death. The AgNPs were observed to exhibit inhibition, and it was inferred that the AgNPs produce reactive oxygen species (ROS) in the nutrient broth that led to the growth inhibition of bacteria. There is evidence that time contributes to the suppression and growth of bacteria. The growth time and lag phase for bacteria are dependent upon the characteristics and functional participation of nanoparticles, including the conditions necessary for bacterial growth and development [51,52].

### 3.9. Antioxidant Potentials

**Figure 14:**
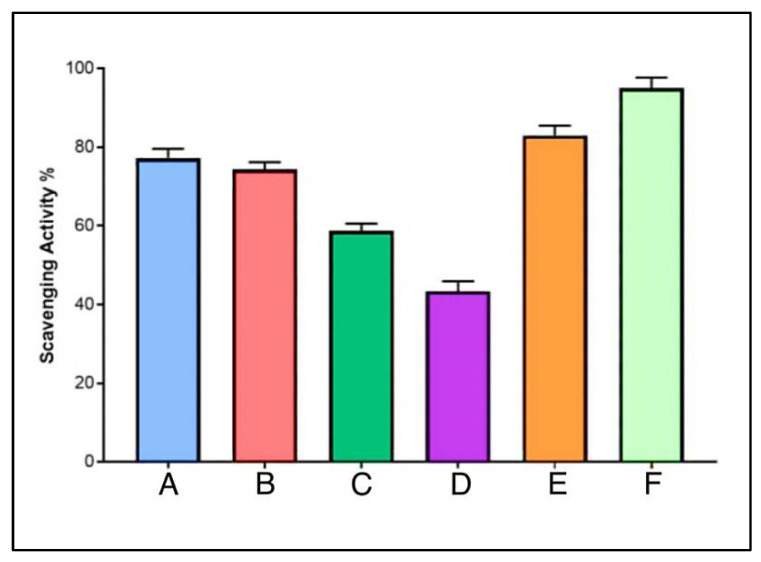
Anti-oxidant potentials of AgNPs at different concentrations (A. 100, B. 50, C. 25, and D. 12.5 μg /mL), E. AgNPs–lincomycin mixture, and F. ascorbic acid.

DPPH, stable at room temperature, displays a dark violet color when dissolved in organic solvent and shows a strong absorption wavelength at 517 nm. With the presence of AgNPs, the color change occurs, which was observed to be yellow. The DPPH is also known to have reduced in the presence of phenolic OH groups [53]. The DPPH assay showed that the AgNPs have antioxidant properties, which were proportionally increased with an increase in the concentration. The AgNPs–lincomycin mixture showed slightly increased antioxidant activity as compared to the AgNPs at 100 μg/mL concentration, the concentration at which the anti-bacterial activity of the AgNPs alone was at its best. The AgNP antioxidant activity, measured as the scavenging potential as a percent scale, in comparison to the ascorbic acid, was at 45, 63, 78 and 80% for the AgNPs concentration at 12.5, 25, 50 and 100 μg/mL, respectively (Figure 14). The antioxidant potential of the AgNPs and the lincomycin mixture was also slightly above the antioxidant potential of the AgNPs alone at the same concentration levels of the AgNPs, thereby suggesting the limited role of only the lincomycin antibiotic’s antioxidant potential, and presumably the need for higher anti-oxidant value to overcome the bacterial drug resistance by the lincomycin. Further work in this direction may shed more light on the phenomenon, and studies are planned for the future.

### 3.10. Genes Expression Levels

**Figure 15:**
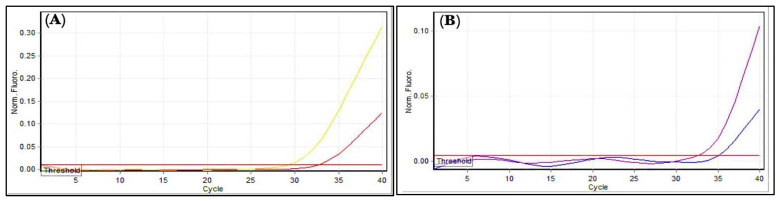
Gene expressions: (**A**) *Bla* gene; (**B**) *Bla_CTX-M-15_*. Amplification plots by qPCR samples before and after treatment with AgNPs. The picture was taken directly from the Cepheid (smart cycler) qPCR.

Total RNA, before and after treatment with AgNPs, was successfully extracted from each bacterial isolate, and purity was measured at wavelengths 260 and 280 nm. The A260/A280 ratio, which was 2, indicated that the RNA was pure, while the concentration ranged from 40 to 80 ng/µL. The expression of *Bla* and *Bla_CTX-M-15_* genes conferred resistance to penicillin in *B. cereus* and *P. mirabilis*, respectively, and these were assessed with and without exposure to the AgNPs. In AgNPs’ presence, the expression of *Bla* and *Bla_CTX-M-15_* genes were decreased by 0.0915 and 0.1768 folds, respectively, as compared with the untreated control group bacteria (Table 7 and Table 8, and Figure 15). The AgNPs seem to influence the antibiotic-resistance genes of bacteria, as they can affect the DNA strand and alter the gene expression. According to earlier reports, the AgNPs cause increased production of reactive oxygen species (ROS). Moreover, unlike DNA, RNA is highly sensitive to oxidative damage caused by ROS. Without RNA-repair mechanisms, fundamental functions such as the control of transcriptional activity may be hampered. As for the mechanism of action of the AgNPs on Bla, *Bla_CTX-M-15_*, the *Bla* genes are known to encode functional β-lactamases that provide the microbes antibiotic resistance by breaking the invading antibiotic’s structure. Numerous studies have confirmed the production of extended-spectrum β-lactamases (ESBLs) by *Proteus* spp. and certain *Bacillus* spp. that hydrolyzes the penicillin class of antibiotics. However, there are no studies available on the effects of AgNPs on the gene expressions of the ESBL-encoding genes in these microbial species. Therefore, the present study could be the latest regarding the ESBLs enzyme restriction, and it concluded that the AgNPs were active against *B. cereus* and *P. mirabilis*, and the expression level of the *Bla* and *Bla_CTX-M-15_* genes, respectively. The expression levels were found to be low after microbial inoculates were treated with AgNPs [2,54].

Thus, the freshly prepared AgNPs, considered better for anti-oxidant potential, were utilized in preparing the combined delivery formulation of the AgNPs and the lincomycin. The anti-bacterial and anti-biofilm forming activities, together with the DPPH scavenging potential, the comparative anti-bacterial and anti-oxidant activities of the AgNPs, and the AgNPs–lincomycin mixture were observed to infer a combined additive action of the lincomycin–AgNPs mixture. The gene expression results agreed with other findings wherein the low expression levels of *Bla* family genes inhibited β-lactamases actions and dispelled the resistance towards penicillin [55,56,57].

## 4. Conclusions

This study’s findings establish that the synthesis of AgNPs using the chemical reduction method provided relatively stable NPs, which were characterized by their physico-chemical properties of size, shape, and chemical compositions. AgNPs–lincomycin as a homogenized mixture showed strong anti-oxidant potential, produced the highest anti-bacterial activity, and showed strong and requisite anti-biofilm formation ability against the *B. cereus* and *P. mirabilis* microorganisms, which helped to overcome the drug resistance of these bacteria against the lincomycin antibiotic. The expression levels of the selected genes, *Bla* and *Bla_CTX-15_* have decreased in *B. cereus* and *P. mirabilis* after treatments with the AgNPs. The observations suggested the mutagenic effect and genotoxic nature of the AgNPs against these bacteria, which may have caused genetic material damage, although this needs to be investigated in detail. The effects on the anti-bacterial activity can be related to both the direct impact of the AgNPs, as well as the indirect effects through the antioxidant potential of the AgNPs in contributing to overcoming bacterial drug resistance.

## Figures and Tables

**Table 1:** Steps of the thermal cycles for cDNA reverse transcriptions.

	Step 1	Step 2	Step 3
Temperature	25 °C	42 °C	85 °C
Time	10 min	15 min	5 s

**Table 2:** Thermal profile of *Bla* and *Bla_CTX-M-15_* genes expressions.

Step	Temperatures (°C)	Time (s)	Cycles
Enzyme activation	94	30	1
Denaturation	94	5	35
Annealing	52	15	
Extension	72	20	

**Table 3:** Bacterial resistance tests of different antibiotics and antibiotics–AgNPs mixture.

Antibiotic	Concentration	*Bacillus cereus*			*Proteus mirabilis*		
	µg/Disk	* Antibiotic	* Antibiotic + AgNPs	** F. I.%	Antibiotic	Antibiotic + AgNPs	F. I.%
AK	10	-	-	-	6	24	300
AMC-30	20/10	6	12	100	14	18	28.5
CTX	30	-	-	-	38	38	-
E	15	24	28	16.7	-	-	-
MY	15	24	34	41.6	6	13	116.7
P	10 IU	6	12	100	-	-	-
TE	30	18	22	22.22	-	-	-
STX	25	-	-	-	24	24	-

* Inhibition zone in (mm), ** F.I.: fold increase, F = ((b − a)/a) × 100; Note: To calculate the fold increase in the absence of bacterial growth inhibition zones, the disc diameter (6 mm) was used.

**Table 4:** Anti-bacterial activity of lincomycin, AgNPs, and antibiotic and AgNPs mixture against bacterial isolates.

Microorganism	AgNPs, 100 μg/mL	AgNPs, 50 μg/mL	AgNPs, 25 μg/mL	AgNPs, 12.5 μg/mL	Lincomycin, 15 µg/mL	AgNPs–Lincomycin, (100 µg/mL + 15 µg/mL)
*B. cereus*	17.50 ± 0.50	14.00 ± 1.00	11.50 ± 0.5	11.00 ± 0.5	24.33 ± 0.5	35.33 ± 0.50
*P. mirabilis*	26.00 ± 0.50	25.00 ± 0.50	17.33 ± 0.50	14.00 ± 0.50	6.00 ± 0.00	21.66 ± 1.00

**Table 5:** MIC and MBC of the bacterial isolates.

Bacteria	Conc. of AgNPs (μg/mL)		Conc. of AgNPs–Lincomycin (μg/mL)	
	MIC	MBC	MIC	MBC
*B. cereus*	50	100	12.5	25
*P. mirabilis*	50	100	25	50

**Table 6:** Growth curve assays of AgNPs against *B. cereus* and *P. mirabilis*.

Bacteria	AgNPs (100 μg/mL)			
	Zero	30 min	60 min	90 min
*B. cereus*	*++++*	*+++*	*++*	*+*
*P. mirabilis*	*++++*	*+++*	*++*	*+*

Note: (++++) Very dense growth, (+++) Dense growth, (++) Medium growth, (+) Few growth.

**Table 7:** Comparison of *Bla* gene’s fold expressions between the groups.

Groups	Mean Ct, *Bla*	Mean Ct, HKG	ΔCt (Mean Ct, *Bla*—Mean Ct, HKG)	2^−ΔCt^	Experimental Group/Control Group	Fold(s) of Gene Expression
Group # 1 (Control)	29.2	22.1	7.1000	0.0073	0.0073/0.0073	1.0000
Group # 2 (After AgNPs treatment)	32.65	22.1	10.5500	0.0007	0.0007/0.0073	0.0915

**Table 8:** Comparison of *Bla_CTX-M-15_* gene’s fold expression between groups.

Groups	Mean Ct, *Bla_CTX-M-15_*	Mean Ct, HKG	ΔCt (Mean Ct, *Bla_CTX-M-15_*—Mean Ct, HKG)	2^−ΔCt^	Experimental Group/Control Group	Fold of Gene Expression
Group # 1 (Control)	32.58	18.9	13.6800	0.00008	0.00008/0.00008	1.0000
Group # 2 (After AgNPs treatment)	35.08	18.9	16.1800	0.000013	0.000013/0.00008	0.1768

## Data Availability

All data are provided in the manuscript.
